# A Discourse on Self Limited Diseases

**Published:** 1835

**Authors:** 


### Art. IX.—Self-Limited Diseases.

*Discourse on Self-Limited Diseases, delivered before the Massachusetts Medical Society, at their Annual Meeting, May* 27, 1835. *By Jacob Bigelow, M. D. Professor in Harvard University.* pp. 48.

The object of this neat little discourse, is to make a recognition, and attempt a classification, of those diseases which, in despite of any method of treatment hitherto discovered, are known to run through certain *stadia*, when they either prove fatal, or cease, and the patient is restored to health—though sometimes more or less scarred, or permanently altered in the structure of the affected parts. The existence of such maladies has long been perceived, and they have, generally, been held to be incurable. We have, however, heard a physician, of the Broussais school, affirm that by the antiphlogistic treatment he had arrested the small pox, and caused it to abort—that is, prevented the eruption and the secondary fever. That the extent and intensity of these stages may be much reduced, has been known ever since the cold treatment was introduced; but we entertain serious doubts of the possibility of terminating the disease, after the contagion has taken effect. Should this and the other "self-limited" diseases, as Prof. Bigelow styles them, ever be cut short, it will, we predict, be done by other agents than those which are purely antiphlogistic—by means which establish in the system such an altered state of the vital properties, as is incompatible with the continuance or the spread of the morbid actions previously set up. To the efficacy of such agents the predisposing influence of depletion may be important—sometimes indispensable, as we observe that the cinchona will not cure intermittent fever, nor the mercurial preparations, syphilis, at least with any degree of certainty, or comfort to the patient, if there be plethora and a high tone of inflammatory action; while they never fail after due depletion.

"The class of diseases under consideration, comprehends morbid affections, differing greatly from each other, in the time, place and nature of their spontaneous developments; so that they may admit of at least three general subdivisions. These may be called, 1st. The *simple;* in which the disease observes a continuous time, and mostly a definite seat: 2d. The *paroxysmal;* in which the disease, having apparently disappeared, returns at its own periods: and, 3d. The *metastatic;* in which the disease undergoes metastasis or spontaneous change of place. In the present state of our knowledge, we have no difficulty in finding examples of each of these subdivisions. There are also other examples, in which the disease, although capable of being in part influenced by medical treatment, still retains a portion of its original intractability, and has strong relations to the class in question." p. 11.

Under the first head, Dr. B. arranges *hooping-cough*, *measles*, *scarlatina*, *small pox*, *erysiplas*, and doubtingly, *typhus*. From his observations on *scarlatina* we extract with approbation the following sentence:—

"The modes of treatment, which have had most testimony in their favor, are various, and opposite. By Dr. Fothergill, stimulants were relied on; by Dr. Currie, cold water; by Dr. Southwood Smith, and others, blood-letting. But it is not satisfactorily shown, that either of these modes of practice has been particularly successful; for where the writers have furnished us any thing like definite, or numerical results it does not appear that the mortality was less in their hands than it is among those who pursue a more expectant practice. The post mortuary appearances, which in many diseases furnish useful lessons for practice, are in scarlet fever extremely various and uncertain; and sometimes no morbid changes, sufficient to account for death, can be discovered in any of the vital organs, or great cavities." p. 14.

Erysipelas is not generally regarded as a self-limited disease, but every physician must have met with cases in which the inflammation could not be arrested till it reached the extremity of the limb or member on which it appeared. Thus, commencing on each temple, we have seen it deliberately advance forward, resisting all constitutional and local treatment, till it passed off at the tip of the nose.

The self-limited character of typhus is more doubtful. The period of its duration is exceedingly various; but this may perhaps arise from the varying intestity of action of the remote cause. Our author very strongly inclines to the conclusion, that typhus cannot be shortened, and even doubts whether it can be broken up in its forming stage. For ourselves, we are compelled to confess, that we have seldom seen this disease more than mitigated, by any plan of treatment, to which it has been subjected.

"We come next to a second order of self-limited diseases, of which the term *paroxysmal* is sufficiently descriptive. This term applies to certain morbid affections, which recur in fits or paroxysms, leaving the patient comparatively well in the intervals, at the same time that the paroxysms themselves can neither be foreseen, prevented, nor, as far as we know, materially abridged in their duration." p. 21.

Under this division is classed *epilipsy*, *angina pectoris*, formerly *gout*, *mania*, *melancholy*, *asthma*, *gravel*, and *ascarides* in the rectum.

"Cases, which bear the names of all the above diseases, are undoubtedly relieved, and sometimes even removed by medicine; but it is equally true, that other cases are wholly intractable, both as to their recurrence, their duration, and their susceptibility of much change from medical treatment. And it will come to the recollection of many practitioners, that they have, in the course of their lives, believed themselves to have cured these diseases, when in fact they have only witnessed the spontaneous subsidence of a paroxysm." p. 23.

Our author's third general head embraces *metastatic* affections, of which he enumerates several.

"By this term I wish to express certain morbid affections, which pass by metastasis from one part of the body to another, for the most part independently of artificial influence. Of this kind are certain *cutaneous* affections, more especially some which are chronic and hereditary. Many persons pass a considerable portion of their lives in alternate annoyance from a disease of the skin, and from its vicarious substitute in some internal organ. Others again are afflicted with hemorrhagic, or purulent *discharges*, which at times disappear, only to be succeeded by equally troublesome affections in a different part. *Gonorrhœa* cannot be prevented from occasional metastasis of inflammation to the testes, and *mumps* are sometimes found to undergo the same transition. But perhaps the most remarkable example of a metastatic disease is found in *acute rheumatism*. This morbid affection often begins to discover itself in a limited and comparatively unimportant part of the system. From thence, in grave cases, it travels by successive migrations from joint to joint, and from limb to limb, till it has visited nearly all the great articulations of the body. It also attacks the organs of sense, and the viscera which are essential to life. During the course of these migrations, the attending physician cannot foretell at any given stage, what part will be next invaded by the disease, neither can he protect any part from being thus invaded; nor can he control the period, during which the disease will reside in any particular part previously to its next metastasis. And in alarming and dangerous cases of this disease, it often happens that the physician can do little more than stand by with his palliatives, in the anxious hope, that the disease will at length pass by another metastasis, to some less vital, or less important organ." p. 24.

Dr. Bigelow has added to his discourse a variety of instructive notes, one of which is on verminous diseases, with an extract from which we shall close this notice.

"It is commonly believed that the principal residence of ascarides is in the rectum, because they are most felt there. They have been found however, in every part of the alimentary tube. Many patients, immediately after a cessation of the annoyance in the rectum, are visited by pain in the epigastrium, attended with costiveness and clay-colored discharges. This state continues for two or three days, and is then followed by a bilious diarrhea. I have repeatedly known these consecutive events to occur with great regularity for half a dozen years, so much so that my inquiries are generally directed towards this cause, when children have complained of epigastric pains at regular periods. Whether, in these cases, the worms ascend to the duodenum and mouth of the biliary duct, or whether the whole is an affair of sympathy, future autopsis may perhaps determine." p. 44.

